# Data for quantum-chemical modeling of the mechanisms of ring-opening polymerization of methyl ethylene phosphate

**DOI:** 10.1016/j.dib.2019.104431

**Published:** 2019-08-29

**Authors:** Ilya E. Nifant'ev, Andrey V. Shlyakhtin, Maxim A. Kosarev, Pavel D. Komarov, Stanislav G. Karchevsky, Pavel V. Ivchenko

**Affiliations:** a M.V. Lomonosov Moscow State University, Department of Chemistry, Leninskie Gory 1–3, Moscow, 119991, Russia; b A.V. Topchiev Institute of Petrochemical Synthesis RAS, Leninsky Avenue 29, Moscow, 119991, Russia; c Joint-stock Company "Institute of Petroleum Refining and Petrochemistry", Iniciativnaya Str. 12, Ufa, 450065, Republic of Bashkortostan, Russia

**Keywords:** DFT, Ring-opening polymerization, Ethylene phosphates, Organocatalysis, Coordination catalysis

## Abstract

The data presented in this paper are related to the research article entitled "Mechanistic study of transesterification in TBD-catalyzed ring-opening polymerization of methyl ethylene phosphate" (Nifant'ev et al., 2019). In this data article, we present 3D molecular information of 76 structures for TBD-catalyzed transformations of methyl ethylene phosphate (MeOEP) and trimethyl phosphate (TMP). We also present 3D molecular information for 24 complexes that model the reaction profile of transesterification of poly(MeOEP) and TMP catalyzed by 2,6-di-*tert*-butyl-4-methylphenoxy magnezium species, complementing the article "Mechanistic insights of BHT-Mg-catalyzed ethylene phosphate's coordination ring-opening polymerization: DFT modeling and experimental data" (Nifant'ev et al., 2018). The data contains stationary points and transition states (TS) along the first propagation step of MeOEP ring-opening polymerization (ROP) for alternative amide and donor-acceptor mechanisms, initiated by EtOH in the presence of TBD; stationary points and TS for MeOH and HOCH_2_CH_2_OP(O)(OMe)_2_ initiated ROP of MeOEP; and stationary points and TS for transesterification of poly(MeOEP) and TMP. In addition, the data contains stationary points and transition states for the ROP of MeOEP and transesterification of poly(MeOEP) and TMP catalyzed by 2,6-di-*tert*-butylphenoxy magnesium complex. The data are provided in a PDB format that can be used for further studies.

**Table:** Specifications Table

Subject area	Chemistry
More specific subject area	Physical and Theoretical Chemistry Polymer chemistry
Type of data	PDB files
How data was acquired	Density functional theory calculations with Gaussian 09
Data format	Raw
Experimental factors	The dataset of 100 molecular structures were generated from density functional theory (DFT) calculations
Experimental features	Geometry optimization with B3PW91 functional. Effective core potential DGTZVP was used.
Data source location	Moscow, Russian Federation
Data accessibility	Data are supplied with this article
Related research article	I.E. Nifant'ev, A.V. Shlyakhtin, A.N. Tavtorkin, M.A. Kosarev, D.E. Gavrilov, S.O. Ilyin, S.G. Karchevsky, P.V. Ivchenko. Mechanistic study of transesterification in TBD-catalyzed ring-opening polymerization of methyl ethylene phosphate, Eur. Polym. J. 118 (2019) 393–403, https://doi.org/10.1016/j.eurpolymj.2019.06.015.

**Table:** 

**Value of the data** • The PDB coordinates of the stationary points and transition states of ring-opening polymerization (ROP) of methyl ethylene phosphate (MeOEP) represent the first Data set of DFT modeling of the ROP of phosphorus-containing cyclic monomers • Method and basis set employed in this Data article can be used as a reference for future studies • The Data obtained can be effectively applied for the simulation of ROP of other phosphorus-containing cyclic monomers • The Data obtained can be compared with the Data of DFT modeling of the ROP of conventional monomers such as lactides, lactones and cyclic carbonates and may be used for theoretical interpretation and prediction of the synthesis of ester-phosphate copolymers that are highly actual as biocompatible and biodegradable materials for biomedical applications • The Data obtained can be applied for the design of novel effective ROP catalysts by other researches in this field

## 1 Data

The data described in this paper provides in formation for the electronic structures for reaction components of TBD-catalyzed ring-opening polymerization (ROP) of methyl ethylene phosphate, for stationary points and transition states of the ROP of MeOEP initiated by MeOH, EtOH and model macroinitiator HOCH2CH2OP(O)(OMe)_2_, as well as for stationary points and transition states of the transesterification of poly(MeOEP) by using triphosphate MeOP(O)[OCH2CH2OP(O)(OMe)_2_]_2_ as a model of polyphosphate chain, and trimethyl phosphate (TMP) as a low-molecular acyclic phosphate (see Ref. [1] for more details). The data set also includes stationary points and transition states for the ROP and transesterifications in the presence of the model 2,6-di-*tert*-butylphenoxy magnesium (DBP-Mg) coordination catalyst (these Data complement the article [2] which includes the results of the DFT modeling of DBP-Mg-catalyzed polymerization of MeOEP). The data set of 100 structures were generated from density functional theory (DFT) calculations [3], [4]. Atomic coordinate files for each species of all reagents and catalysts are provided in PDB format in the Supplementary material.

## 2 Experimental design, materials, and methods

All geometries were fully optimized at using Gaussian 09 program [5]. The B3PW91 hybrid functional [6], [7] and DGTZVP basis [8], [9] were applied for calculations. The applicability of B3PW91 functional for the modeling of ring-opening polymerization was described earlier [2], [10], [11], [12], [13], [14], [15]. The optimization of stationary points geometries, frequency analysis, and calculations of entropy corrections were made for gas phase at 298.15 K. Transition states were found directly by Berny optimization and confirmed by the relaxation to corresponding stationary structures after changing of key geometric parameter with a step of ±0.01 Å.
